# Case report: Acute unintentional carbachol intoxication

**DOI:** 10.1186/cc4937

**Published:** 2006-06-01

**Authors:** Martin Schulz, Thomas Graefe, Klaus Stuby, Hilke Andresen, Nikolai Kupfermann, Achim Schmoldt

**Affiliations:** 1 Department of Pharmacology, University of Frankfurt, and Head, Center for Drug Information and Pharmacy Practice, ABDA – Federal Union of German Associations of Pharmacists, Jaegerstrasse 49/50, 10117 Berlin, Germany; 2 Department of Internal Medicine, Hospital Pirmasens, 66955 Pirmasens, Germany; 3 Toxicology Unit, Institute of Legal Medicine, University of Hamburg, 22529 Hamburg, Germany

## Abstract

**Introduction:**

Intoxications with carbachol, a muscarinic cholinergic receptor agonist are rare. We report an interesting case investigating a (near) fatal poisoning.

**Methods:**

The son of an 84-year-old male discovered a newspaper report stating clinical success with plant extracts in Alzheimer's disease. The mode of action was said to be comparable to that of the synthetic compound 'carbamylcholin'; that is, carbachol. He bought 25 g of carbachol as pure substance in a pharmacy, and the father was administered 400 to 500 mg. Carbachol concentrations in serum and urine on day 1 and 2 of hospital admission were analysed by HPLC-mass spectrometry.

**Results:**

Minutes after oral administration, the patient developed nausea, sweating and hypotension, and finally collapsed. Bradycardia, cholinergic symptoms and asystole occurred. Initial cardiopulmonary resuscitation and immediate treatment with adrenaline (epinephrine), atropine and furosemide was successful. On hospital admission, blood pressure of the intubated, bradyarrhythmic patient was 100/65 mmHg. Further signs were hyperhidrosis, hypersalivation, bronchorrhoea, and severe miosis; the electrocardiographic finding was atrio-ventricular dissociation. High doses of atropine (up to 50 mg per 24 hours), adrenaline and dopamine were necessary. The patient was extubated 1 week later. However, increased dyspnoea and bronchospasm necessitated reintubation. Respiratory insufficiency was further worsened by *Proteus mirabilis *infection and severe bronchoconstriction. One week later, the patient was again extubated and 3 days later was transferred to a peripheral ward. On the next day he died, probably as a result of heart failure. Serum samples from the first and second days contained 3.6 and 1.9 mg/l carbachol, respectively. The corresponding urine concentrations amounted to 374 and 554 mg/l.

**Conclusion:**

This case started with a media report in a popular newspaper, initiated by published, peer-reviewed research on herbals, and involved human failure in a case history, medical examination and clinical treatment. For the first time, an analytical method for the determination of carbachol in plasma and urine has been developed. The analysed carbachol concentration exceeded the supposed serum level resulting from a therapeutic dose by a factor of 130 to 260. Especially in old patients, intensivists should consider intoxications (with cholinergics) as a cause of acute cardiovascular failure.

## Introduction

Carbachol is a muscarinic cholinergic receptor agonist discovered in 1932 [1]. It retains substantial nicotinic activity, particularly on autonomic ganglia. It is likely that both its peripheral and its ganglionic actions are due, at least in part, to the release of endogenous acetylcholine from the terminals of cholinergic fibres [2]. Its clinical use in the treatment of urinary bladder disorders or gastrointestinal atony has, however, diminished. Today, carbachol (1 to 3%) is mainly used as eye drops to lower intraocular pressure in glaucoma.

We report an interesting case of an accidental carbachol ingestion leading to massive intoxication.

## Case report

The son of an 84-year-old male discovered a media report in Germany's most popular daily newspaper stating clinical success with plant extracts (*Salvia lavandulaefolia *(Spanish sage) and *Melissa officinalis *(lemon balm), among others) in Alzheimer's disease. According to this report, the mode of action of these extracts are said to be comparable to the synthetic compound 'carbamylcholin'; that is, carbachol.

Approaching a pharmacy for a medicine containing this drug, he was informed that all such brand products were prescription-only. However, he was told that this holds true only for the registered medicines but not for the substance. Eventually, he ordered and bought 25 g of carbachol as pure substance. Eleven days later, the patient was administered a teaspoon equivalent to 400 to 500 mg of carbachol (the average parenteral therapeutic dose is 1 mg). Minutes later, he developed nausea, sweating and hypotension, and finally collapsed. The emergency physician observed bradycardia and cholinergic symptoms. Some minutes later asystole occurred. The physician initiated cardiopulmonary resuscitation, immediately injecting 3 × 1 mg of adrenaline (epinephrine), 2 × 0.5 mg of atropine and 80 mg of furosemide (at that time, cholinergic/carbachol intoxication was not diagnosed; the rationale for administering furosemide was suspicion of left ventricular failure). He was intubated and ventilated, and cardiac output returned.

On hospital admission, 0.5 mg of atropine was injected intravenously and 60 g of activated charcoal as well as 60 ml of lactulose were administered through a nasogastric tube (although diarrhoea might be expected after cholinergic poisoning, a laxative was administered to avoid constipation and hence prolonged absorption of carbachol as a result of atropine therapy). On the intensive care unit, the blood pressure of the bradyarrhythmic patient was 100/65 mmHg and the heart rate was 60 beats/minute. Further signs were hyperhidrosis, hypersalivation, bronchorrhoea, and severe miosis; the electrocardiographic finding was atrio-ventricular dissociation. The remaining physical examination was unremarkable.

On the first day, a total of 50 mg atropine (2 mg/hour) and a perfusion of adrenaline and dopamine were necessary.

Significant laboratory test results were as follows: leukocyte count (WBC) 14.4 × 10^9^/l (peaking at 22.0 × 10^9^/l), platelet count 250 × 10^9^/l, haemoglobin 12.5 g/dl (trough at 11.0 g/dl), serum creatinine 221 μmol/l (peaking at 292 μmol/l), urea 33.3 mmol/l, total protein 55 g/l, C-reactive protein 35 mg/l (peaking at 208 mg/l), and blood glucose 12.5 mmol/l.

Chest X-ray on admission and during the first part of the treatment showed no signs of inhalation pneumonia.

After administration of decreasing doses of atropine, adrenaline and dopamine, the patient was extubated 1 week later. However, increasing dyspnea and bronchospasm necessitated reintubation. A bronchopulmonary infection, identified in the tracheal secretion as *Proteus mirabilis*, was treated with ampicillin/sulbactam, severe bronchoconstriction with salbutamol, theophylline, and high doses of prednisolone.

One week later, the patient was again extubated and three days later transferred to a peripheral ward. On the next day he died. As the respiratory situation was considerably improved and sepsis signs were not observed, death was most probably related to heart failure.

Known diseases were Alzheimer's disease, heart failure and diabetes type 2.

Ambulatory care medication was piracetam, furosemide, captopril, metformin and glyburide.

We obtained written consent and permission to publish this case, signed by the son of the patient.

## Analytical method

Carbachol (INN; 2-carbamoyloxyethyl)trimethylammonium chloride; IUPAC, *M*_r _182.65) is a strong base containing a quaternary nitrogen atom, usually not extractable with organic solvents and showing no characteristic ultraviolet absorption. In addition, gas-chromatographic quantification of this compound is not possible. To the best of our knowledge, levels of carbachol in blood, serum or urine in humans have never been analysed. Both pharmacokinetic and toxicokinetic data of carbachol in human are therefore generally lacking. Hence, a specific method for the determination of carbachol in human blood and urine by HPLC-mass spectrometry (liquid chromatography – tandem mass spectrometry (LC-MS/MS)) was developed: to 0.1 ml of serum, 0.8 ml of bidest water, 0.1 ml of 2.5% ammonia and 0.001 mg of triethylbenzylamine as the internal standard were added. After centrifugation (1 minute at 10,000 *g*), the supernatant was extracted by solid-phase extraction with the use of cation-exchange columns (Bond Elut^® ^CBA; Varian, Darmstadt, Germany). The cartridges were conditioned with 2 × 1 ml of methanol and 2 × 1 ml of water, followed by loading with the supernatant. After rinsing sequentially with 1 ml of water, 1 ml of methanol and 1 ml of water, elution was performed with 1 ml of 0.05 mol/l HCl; 0.01 ml of the eluate was injected into the LC-MS/MS system (LCQ DUO; Thermo Finnigan, Egelsbach, Germany). HPLC was performed with a 2 mm × 15 cm C_18 _reverse-phase silica column (SymmetryShield^®^; Waters, Eschborn, Germany). The mobile phase used was 0.001 mol/l formic acid at a flow rate of 0.1 ml/minute. For validation, calibration curves with spiked plasma were analysed and were linear over a concentration range of 0.5 to 7.0 mg/l carbachol (*r *= 0.993), and the limit of quantification was less than 0.02 mg/l.

Peaks of carbachol (*m*/*z *147) and internal standard (*m*/*z *192) were detected and identified by electrospray positive ionisation (+cESI) MS/MS and quantified by integration of fragment ions at *m*/*z *60 + 88 and *m*/*z *91 + 100, respectively.

For urine analysis, samples could be used without solid-phase extraction but had to be diluted 1:1,000. After the addition of appropriate amounts of internal standard, LC-MS/MS was performed as described for serum.

### Analytical results

Serum samples of the first and second days of hospital admission contained 3.6 and 1.9 mg/l carbachol, respectively. The corresponding urine concentration amounted to 374 and 554 mg/l.

## Discussion

Intoxications with carbachol are rare. The first was described in 1944: a man died after injection of an ampoule containing carbachol in crystal form not intended for internal use [3].

A 10-year-old boy was fatally poisoned by his mother, who added 15 to 20 mg of carbachol to his soup several times. Her 36-year-old husband was treated because of life-threatening attacks of profuse sweating, intestinal cramps, explosive defecation, hypothermia, hypotension and bradycardia. From that case, a lethal dose in children of 1 mg/kg has been suggested [4]. The lethal dose in mice after oral administration is 0.25 mg/kg [1].

After subcutaneous injection of a therapeutic dose, a patient developed vomitus, severe central chest pain, increasing breathlessness and eventually esophageal rupture. He died 24 days later [5].

Even administering one or two eye drops containing 2 to 3% carbachol can result in severe systemic adverse effects resulting from absorption in the nasolacrimal duct [6,7].

Assuming a maximum volume of distribution of 1 l/kg, a parenteral administered therapeutic dose of 1 mg of carbachol would have resulted in a serum concentration of about 0.014 mg/l. The analysed concentration in this case exceeded the supposed serum level resulting from a therapeutic dose by a factor of 130 to 260. This might explain the continuous bronchoconstriction and possibly neuromuscular insufficiency even 1 week after extubation.

Dispensing carbachol as a pure substance in a community pharmacy to a customer seems unbelievable. Eventually, the charge by the investigating agency (police and prosecution) was dropped because the individual person in the pharmacy dispensing carbachol to the patient's son could not be identified and the supervising pharmacist was fined.

It is remarkable how thoughtless and irresponsible journalists are in reporting on research in the lay press, taking the cholinergic effects of high concentrations of herbal extracts *in vitro *[8] and postulating the clinical benefit in Alzheimer's disease. Comparing the mode of action with 'carbamylcholin' prompted the son to seek this medicine to help his father.

Finally, researchers, peer reviewers, and publishers need to be reminded of their responsibilities before extrapolating laboratory findings and suggesting the use of herbals in a disease such as dementia [9-11]. Today, such publications [10] are easily available through the Internet [12].

## Conclusion

We describe a case of an unintentional carbachol intoxication in an 84-year-old male patient with known heart failure and diabetes, and at an advanced stage of Alzheimer's disease. Although initial cardiopulmonary resuscitation was successful, the patient presented severe persistent cholinergic symptoms complicated by respiratory infection. He eventually died. For the first time, carbachol serum and urine concentrations were analysed by HPLC-mass spectrometry, and the serum level was found to exceed the supposed level resulting from a therapeutic dose by a factor of 130 to 260. Especially in old patients, emergency and intensive care physicians should consider intoxications (with cholinergics) as a cause of acute cardiovascular failure.

## Key messages

• 	A case of an unintentional, acute, severe and eventually fatal intoxication with carbachol in an 84-year-old male with advanced Alzheimer's disease is presented.

• 	Especially in old patients, intoxications (with cholinergics) should be considered as a cause of acute cardiovascular failure.

• 	Prolonged administration of high doses of atropine is necessary to control severe cholinergic symptoms.

• 	For the first time, a sensitive HPLC-mass spectrometry method to analyse carbachol serum and urine concentrations in humans has been developed.

• 	Researchers, peer reviewers and publishers need to be reminded of their responsibilities before extrapolating laboratory findings and suggesting therapeutic opportunities for a disease such as dementia.

## Abbreviations

HPLC = high-performance liquid chromatography; LC-MS/MS, liquid chromatography – tandem mass spectrometry.

## Competing interests

The authors declare that they have no competing interests.

## Authors' contributions

MS had the principal idea and participated in the initial set-up of the study, analytical procedures, literature search, writing, carefully reading, discussing, revising, and approving the manuscript. He approved the final version of the manuscript and is the guarantor. TG and KS were responsible for the clinical examination, treatment and clinical report of the patient. Both discussed and approved the final version of the manuscript. HA and NK participated in the study with regard to developing the method and the corresponding analytical procedures and the validation of carbachol analysis in human serum and urine. Both discussed and approved the final version of the manuscript. AS participated in the initial set-up of the study, analytical procedures, writing, discussing, revising and approving the manuscript. He saw and approved the final version of the manuscript.
